# Bisphosphonate-Related Osteonecrosis of the Jaw: A 10-Year Analysis of Risk Factors and Clinical Outcomes

**DOI:** 10.3390/jcm14134445

**Published:** 2025-06-23

**Authors:** Carmen Gabriela Stelea, Emilia Bologa, Otilia Boișteanu, Alexandra-Lorina Platon, Șerban-Ovidiu Stelea, Gabriela Luminița Gelețu, Cezara Andreea Onică, Daniela Șulea, Mihai-Liviu Ciofu, Victor Vlad Costan

**Affiliations:** 1 Department of Surgery, Faculty of Dental Medicine, “Grigore T. Popa”, 16 Universitatii Str., 700115 Iasi, Romania; carmen.stelea@umfiasi.ro (C.G.S.); stelea_serban-ovidiu@d.umfiasi.ro (Ș.-O.S.); gabriela.geletu@umfiasi.ro (G.L.G.); cezara-andreea.onica@umfiasi.ro (C.A.O.); suleadaniela@gmail.com (D.Ș.); mihai.ciofu@umfiasi.ro (M.-L.C.); victor.costan@umfiasi.ro (V.V.C.); 2 Department of Orthodontics and Dentofacial Orthopaedics, Faculty of Dental Medicine, “Grigore T. Popa” University of Medicine and Pharmacy, 16 Universitatii Str., 700115 Iasi, Romania; alexandra.lorina.platon@umfiasi.ro

**Keywords:** bisphosphonates, osteonecrosis of the jaw, risk factors, hypertension, diabetes

## Abstract

**Background**: Bisphosphonate-related osteonecrosis of the jaw (BRONJ) represents a severe complication associated with bisphosphonate therapy commonly used in patients with osteoporosis and malignancies. **Methods**: This retrospective study evaluates the risk factors and clinical outcomes of BRONJ patients treated at the Oral and Maxillofacial Surgery Clinic in Iaşi, Romania, with the goal of optimizing preventive and therapeutic strategies. Data from 72 BRONJ patients treated between January 2013 and December 2023 were analyzed. **Results**: The majority (83.3%) of patients had underlying malignancies, predominantly breast and prostate cancers. The mandible was most affected, with tooth extraction identified as the primary triggering event. Systemic comorbidities, notably arterial hypertension, diabetes mellitus, and concurrent chemotherapy, were significantly associated with increased BRONJ severity. Surgical intervention was frequently required, with sequestrectomy being the predominant procedure, reflecting advanced disease at the time of diagnosis. **Conclusions**: The findings underline the critical importance of early identification, preventive dental management, and a collaborative multidisciplinary approach to improve patient prognosis.

## 1. Introduction

BRONJ was first reported in 2003 when cancer patients receiving bisphosphonate therapy developed jawbone necrosis [1]. Further research subsequently recognized similar jawbone necrosis associated with additional medications, including denosumab and antiangiogenic agents, leading to the broader classification termed medication-related osteonecrosis of the jaw (MRONJ) [2]. According to the latest AAOMS update, MRONJ is defined as exposed bone in the maxillofacial region that persists for over eight weeks in patients treated with antiresorptive or antiangiogenic agents, and without a history of craniofacial radiation therapy [3]. Therefore, MRONJ serves as an umbrella term encompassing BRONJ, recognizing the involvement of a wider range of medications with similar pathophysiological mechanisms and clinical presentations [3,4].

Bisphosphonates are widely used to manage osteoporosis and cancer-related bone diseases, particularly metastatic bone lesions in breast cancer, prostate cancer, and multiple myeloma [5]. While these medications effectively reduce skeletal complications, they also impair bone healing and remodeling, increasing BRONJ risk. Bisphosphonates, while effectively inhibiting osteoclast activity and reducing bone resorption, also impair the jawbone’s capacity to heal after trauma or infection, potentially resulting in necrosis [6].

Several risk factors have been identified in the development of BRONJ. These include local factors, such as invasive dental procedures, periodontal disease, and poor oral hygiene, and systemic factors like anemia, diabetes, immunosuppressive therapy, and chemotherapy [7,8]. Tooth extractions remain one of the most common local triggers for BRONJ development [9,10]. Dental trauma or infections that require extraction or other invasive procedures can expose the underlying bone, which, in patients with compromised bone healing due to medication, can lead to necrosis. Systemic conditions like diabetes further complicate the situation. Diabetic patients often have compromised immune function and impaired wound healing, both of which increase the risk of developing BRONJ. Similarly, patients undergoing chemotherapy or immunosuppressive therapies are at a higher risk due to the reduced ability of their bodies to mount a proper healing response [8].

The prevalence of BRONJ varies depending on the population studied and the type of medication involved. In patients receiving oral bisphosphonates for osteoporosis, the incidence of BRONJ is relatively low, estimated at less than 0.1% [5]. In contrast, among cancer patients receiving intravenous bisphosphonates or denosumab for metastatic bone disease, BRONJ incidence rises significantly, ranging from 1% to 15%, depending on treatment duration and intensity [2].

The management and prevention of BRONJ remain challenging, with no definitive cure available. Prevention strategies focus on early diagnosis and minimizing risk factors before the initiation of bisphosphonate therapy. For instance, dental evaluations and treatments are recommended prior to starting this therapy to reduce the likelihood of invasive procedures, such as tooth extractions, during treatment [11]. Maintaining good oral hygiene and regular dental check-ups are also essential preventive measures. Once BRONJ develops, treatment is typically conservative and aimed at managing symptoms rather than curing the condition. Conservative treatment approaches include antimicrobial mouth rinses, systemic antibiotics, and pain management. In cases where necrotic bone is exposed, surgical debridement may be necessary to remove dead tissue and promote healing, though this approach is used cautiously due to the risk of exacerbating the condition [12].

While tooth extraction has been widely accepted as the main local trigger for MRONJ, it increasingly appears that this association may, in some cases, reflect pre-existing oral pathology that necessitated the extraction rather than the surgical procedure itself. Recent studies suggest that chronic dental infections—such as untreated apical periodontitis, advanced periodontal disease, or pericoronitis—as well as failed endodontic treatments, may act as independent risk factors by maintaining a state of persistent local inflammation and compromising bone integrity [13]. These subclinical or clinically overlooked conditions may create a favorable microenvironment for osteonecrosis, especially in patients receiving high-potency antiresorptive or antiangiogenic therapies. Additionally, structural complications associated with inadequate root canal fillings (e.g., overextension, perforations, undetected root fractures) have been implicated in several MRONJ cases, even in the absence of recent extractions. Despite their potential relevance, the prevalence and etiopathogenic weight of these factors remain underexplored in clinical cohorts, underscoring the need for further real-world data to better characterize their role in MRONJ onset and progression.

This study retrospectively examines BRONJ cases from a clinic in Romania to identify risk factors and epidemiological trends, contributing to the global understanding of this complex condition.

## 2. Materials and Methods

### 2.1. Study Design

This retrospective study was conducted using patient records from the Oral and Maxillofacial Surgery Clinic within the Emergency Clinical Hospital “Sf. Spiridon”, Iași, Romania. Data were collected from January 2013 to December 2023, including demographic details, secondary diagnosis (cancer or osteoporosis), osteonecrosis location, triggering factors, systemic risk factors, and treatment history.

### 2.2. Patients

Patients diagnosed with BRONJ who had received intravenous (IV) zoledronic acid for osteoporosis or cancer were included.

Exclusion criteria encompassed patients treated with other antiresorptive/antiangiogenic agents, those who underwent radiotherapy in the maxillofacial area, and those with oral cancers.

### 2.3. Outcome

The criteria for diagnosing BRONJ, as outlined by the American Association of Oral and Maxillofacial Surgeons (AAOMS) [3], included the following:

(1) Current or previous treatment with bisphosphonates;
(2) The presence of exposed bone persisting for more than eight weeks;
(3) No history of radiotherapy to the jaws or evident metastatic disease in the jaw region.

Patients diagnosed with BRONJ were identified based on the criteria outlined in AAOMS position papers from 2014 and 2022, along with diagnostic codes and data from medical records related to the surgical management of BRONJ at the Oral and Maxillofacial Surgery Clinic within the Emergency Clinical Hospital “Sf. Spiridon”, Iași, Romania. The surgical interventions included bone curettage through sequestrectomy or jaw resection, with the aim of obtaining clinically viable bone.

### 2.4. Data Acquisition

In this study, patient-specific data were extracted from clinical records. Demographic variables collected included sex, age, and place of residence. Systemic risk factors were evaluated by documenting the presence or absence of conditions such as arterial hypertension, diabetes mellitus, obesity, anemia, and prior treatments including chemotherapy, endocrine therapy, immunotherapy, or corticosteroid use. For patients diagnosed with BRONJ, the data encompassed the precise location of the lesion (upper jaw, lower jaw, or both), the identified triggering factor (periodontal disease, periapical infection, or tooth extraction), and the treatment modalities employed (bone resection, sequestrectomy).

### 2.5. Statistical Analysis

Data analysis was performed using Statistical Package for Social Sciences (SPSS), version 20 (IBM Corp., Armonk, NY, USA). The data collected from the patients’ medical charts were initially processed using Microsoft Excel 365 (San Francisco, CA, USA). Differences between groups were assessed using the Chi-square test. Normality tests (Kolmogorov–Smirnov, Shapiro–Wilk) were used to evaluate data distribution. A *p*-value < 0.05 was considered statistically significant.

### 2.6. Ethical Considerations

The study was conducted in accordance with the principles of the Declaration of Helsinki and was approved by the Ethics Committee of the “Grigore T. Popa” University of Medicine and Pharmacy Iași, Romania (approval no. 478/10.07.2024). In addition, all patients signed a general informed consent form upon admission to the “St. Spiridon” Emergency County Clinical Hospital, which authorizes the use of anonymized clinical data for educational and research purposes, in accordance with national regulations (Order 1410/2016 and Law no. 46/2003—The Patients’ Rights Act).

## 3. Results

A total of 72 patients (30 males and 42 females) diagnosed with BRONJ were admitted to the Oral and Maxillofacial Surgery Clinic within the Emergency Clinical Hospital “Sf. Spiridon”, Iași, Romania. The year with the highest numbers was 2014, accounting for 19.4% of the total cases, followed by 2018 with 15.3%. The distribution of cases was relatively even across the other years, indicating consistent occurrence rates. A year-by-year analysis revealed variations in gender and geographical distribution, with a predominance of male patients in 2013 and 2017, whereas female patients were more frequent in 2014, 2015, 2016, and 2020. However, the differences in sex distribution across years were not statistically significant (χ^2^ = 7.77, *p* = 0.651). Additionally, 66.7% of the patients originated from urban environments, suggesting a potential environmental or healthcare accessibility factor. The comparative analysis of geographical origin across study years showed a trend toward urban predominance, though this was not statistically significant (χ^2^ = 16.665, *p* = 0.082).

The mean age of the patients was 65.13 years (SD ± 8.43), with the youngest patient being 37 and the oldest 84 years old. No statistically significant differences in age distribution were observed between genders (χ^2^ = 0.668, *p* = 0.414) or between urban and rural populations (χ^2^ = 0.000, *p* = 1.000), although a slight trend showed that male patients and urban residents tended to be older.

In 83.3% of the cases, the patients had a secondary diagnosis of cancer, with prostate (30.6%) and breast cancers (29.2%) being the most common (Table 1). Osteoporosis was the secondary diagnosis in 15.3% of the cohort.

The majority of osteonecrosis lesions were located in the mandible (65.3%), particularly in the posterior region (72.2%). Dual localization (both maxilla and mandible) was observed in 5.6% of cases, and 4.2% exhibited lesions in both anterior and posterior regions. Localization patterns indicated that the mandibular third and fourth quadrants were most frequently affected.

In 97.2% of cases, BRONJ was triggered by tooth extractions, while 2.8% of patients developed the condition in the presence of non-restorable teeth with chronic pathology, such as periapical lesions, deep caries with pulpal involvement, or advanced periodontal disease. These teeth were considered potential local sources of persistent infection that may have contributed to BRONJ onset, even in the absence of recent extractions. All pathologic teeth were located in the posterior mandibular region. When comparing triggering factors by sex and geographical origin, no significant differences were observed (by sex: χ^2^ = 2.880, *p* = 0.170; by geographical origin: χ^2^ = 1.029, *p* = 0.549).

Regarding surgical treatment, sequestrectomy was performed in 97.2% of cases, while 2.8% of patients underwent partial resection or bone biopsy.

**Figure 1:**
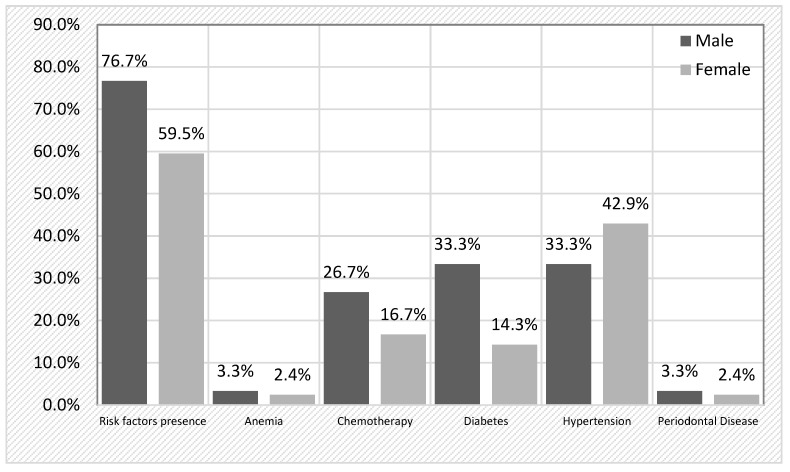
Prevalence of key risk factors in male vs. female BRONJ patients.

A comprehensive analysis of risk factors revealed that 66.7% of the cohort had at least one risk factor for developing BRONJ (Table 2). The most prevalent risk factor was arterial hypertension (38.9%), followed by diabetes (22.2%) and previous chemotherapy (20.8%). Notably, 18% of patients had multiple risk factors, while 48.6% had only one. When stratifying by sex, risk factors were more frequently detected in male patients (76.7%) compared to females (59.5%), although this difference did not reach statistical significance (χ^2^ = 2.314, *p* = 0.128). A detailed breakdown (Figure 1) revealed that chemotherapy was more frequent in men (26.7%) than in women (16.7%) (*p* = 0.303), and diabetes was more common in men (33.3%) compared to women (14.3%) (*p* = 0.055), approaching statistical significance. Hypertension was more prevalent in female patients (42.9%) than in male patients (33.3%).

Similarly, when comparing urban and rural populations, 66.7% of both groups exhibited at least one risk factor. No significant differences were found in the distribution of chemotherapy, diabetes, or hypertension between urban and rural patients (all *p*-values > 0.05).

These results highlight a significant presence of systemic risk factors among BRONJ patients, particularly in males and cancer patients undergoing chemotherapy. Although no statistically significant differences were found, the trends observed suggest a possible impact of systemic health conditions on disease severity.

## 4. Discussion

The findings align with previous studies indicating that BRONJ is predominantly observed in cancer patients receiving bisphosphonate therapy.

The mean age of patients in this cohort was 65.13 years, consistent with findings that suggest older adults are at higher risk for developing BRONJ, primarily due to the increased prevalence of underlying health conditions such as cancer and osteoporosis in this age group [5]. The sex ratio was similar to previous studies, with 58.3% (42 out of 72) of BRONJ cases occurring in females [11,14].

The predominance of BRONJ in patients with a secondary diagnosis of cancer—especially prostate (30.6%) and breast cancers (29.2%)—aligns with existing evidence that antiresorptive agents like bisphosphonates and denosumab are commonly used in the management of metastatic bone disease in these cancers [5,6]. Rugani et al. reported that the prevalence of BRONJ in patients with prostate cancer was higher than for those with breast cancer, but lower compared to patients with multiple myeloma [1]. A longitudinal study offers valuable insights into the impact of comprehensive dental management in patients with multiple myeloma. They found that proactive dental treatment and regular follow-up not only improved patients’ oral health-related quality of life but also significantly decreased the incidence of BRONJ. Their results advocate for early and aggressive dental intervention in high-risk populations, complementing our findings on the importance of preventive dental care in reducing BRONJ occurrence [15].

Khan et al. highlighted that oncology patients with bone metastases experience more intensive osteoclast inhibition than those treated for osteoporosis, leading to a higher incidence of BRONJ. In line with this, our study observed that only 11 out of 72 BRONJ patients had a diagnosis of osteoporosis [16].

The presence of arterial hypertension, diabetes, and chemotherapy as prevalent comorbidities aligns with findings from previous studies, which suggest that these factors may exacerbate the risk of BRONJ through systemic effects on bone metabolism and vascular health [1,6,17]. Although no significant differences were observed between men and women regarding the primary risk factors, the slightly higher prevalence of multiple risk factors in male patients implies that gender may still play a role in the overall risk profile. Hypertension-related vascular dysfunction may reduce blood flow to bone structures, particularly those with a unique vascular supply and a high proportion of cortical bone, such as the mandible. This compromised perfusion may impair the bone’s capacity for healing and its ability to respond to infections, thereby contributing to the pathogenesis of BRONJ. Diabetes mellitus can impair both vascular health and immune function, leading to poor wound healing and an increased susceptibility to infections. Such factors may contribute to BRONJ pathogenesis by impairing the bone’s capacity to recover from minor trauma or infection. Additionally, chemotherapy-induced immunosuppression and direct cytotoxic effects on bone cells may further elevate the risk of osteonecrosis, particularly when combined with antiresorptive therapies [18].

Recent findings underscore that systemic conditions, particularly diabetes mellitus and hypertension, significantly increase the risk of BRONJ in cancer patients undergoing antiresorptive therapy. In their study, nearly 50% of BRONJ cases were associated with these comorbidities, suggesting that chronic inflammation and vascular dysregulation may hinder effective jawbone healing [19]. In parallel, our study also found that a substantial proportion of BRONJ patients presented with diabetes and hypertension, highlighting a consistent pattern across different patient populations. Both studies suggest that impaired wound healing plays a critical role in BRONJ development. These converging findings emphasize the importance of rigorous management of these systemic conditions to potentially reduce the incidence of BRONJ in high-risk groups.

In an Italian study, 413 (91.6%) dentists identified diabetes as an additional risk factor for BRONJ in patients undergoing antiresorptive therapy [20]. Bedogni et al. (2024) also reported that diabetes and hypertension may increase BRONJ risk [21]. Tani et al. (2024) found that diabetes mellitus and numerous bone metastases significantly increase the risk for severe BRONJ, highlighting the importance of vigilant systemic management in these patients [22]. Together, these findings underscore the importance of stringent diabetes management in patients receiving AR therapy to potentially reduce BRONJ risk.

The predominance of osteonecrosis lesions in the mandible (65.3%) corroborates existing literature, which consistently identifies the mandible as the most commonly affected site in BRONJ cases [8]. The notably high incidence of lesions in the posterior region (72.2%) suggests that the unique anatomical and vascular properties of this area may significantly contribute to the pathophysiology of osteonecrosis. These observations are in concordance with other studies, further reinforcing the hypothesis that regional anatomical and vascular factors play a crucial role in the development of BRONJ [23,24].

The fact that 97.2% of cases were triggered by tooth extractions highlights the critical importance of preventive dental care prior to initiating antiresorptive therapies, as emphasized by international guidelines [4]. These findings align with the existing literature, where many guidelines recommend avoiding dentoalveolar surgery whenever possible for patients receiving bisphosphonates [4,16]. However, Otto et al. argue that surgical trauma, such as tooth extraction, is not the primary trigger for BRONJ; instead, local infections are identified as key contributors to its development. They suggest that tooth extraction and other dentoalveolar procedures should not be universally avoided in bisphosphonate-treated patients, particularly when the primary goal is to eliminate a local infection that cannot be treated conservatively. In fact, procedures aimed at addressing and resolving local infections, such as apical or marginal periodontitis, may actually reduce the risk of BRONJ development [9]. Beyond the well-established role of dental extractions, recent evidence has drawn attention to the potential impact of persistent endodontic pathology on MRONJ onset [13]. Although not always clinically symptomatic, these chronic periapical lesions may maintain a pro-inflammatory environment and contribute to subtle bone remodeling disturbances, which could remain undetected in standard dental evaluations. This emphasizes the need for more comprehensive pre-treatment screening protocols in patients scheduled to receive antiresorptive therapies, with a focus not only on surgical history but also on the integrity and status of previously treated teeth.

Recent research provides additional insights into BRONJ risk factors and the role of tooth extractions. Their study found that tooth extractions in patients with malignant tumors receiving high-dose antiresorptive agents significantly reduced BRONJ incidence, whereas no such benefit was observed in osteoporosis patients. Moreover, their data suggest that local infection, rather than the extraction itself, plays a pivotal role in BRONJ development. These findings reinforce the necessity of evaluating dental infection status before determining treatment strategies and highlight the need for proactive dental management to minimize BRONJ risks [25].

Sequestrectomy emerged as the most frequently employed surgical intervention in this cohort, underscoring its critical role in the therapeutic algorithm of advanced BRONJ. While its indication typically corresponds to later disease stages, its widespread use reflects its effectiveness in removing necrotic bone, controlling infection, and facilitating mucosal healing. Rather than indicating therapeutic failure, the high incidence of sequestrectomy highlights its value as a decisive and curative procedure when conservative measures are no longer sufficient. These findings support the integration of sequestrectomy into a structured, stage-based treatment protocol, where timely surgical management contributes significantly to favorable clinical outcomes. Moreover, the data reinforce the importance of multidisciplinary care pathways that combine surgical expertise with systemic disease control to optimize patient prognosis [6,26].

Our findings specifically contribute to existing literature by providing region-specific data from Eastern Europe, enriching the global perspective on BRONJ epidemiology.

The primary limitation of this retrospective study is the reliance on medical records that were not specifically designed for the study’s objectives. Consequently, the collected data may be limited in scope, making it challenging to identify potential confounding factors and increasing the risk of selection bias in patient inclusion. Additionally, the retrospective design introduces several threats to external validity, restricting the interpretation and generalizability of the findings.

## 5. Conclusions

This study highlights the critical role of early detection, preventive dental care, and comprehensive management to reduce BRONJ risks, especially in patients with multiple risk factors. The high incidence of sequestrectomy reflects the advanced stage of BRONJ at presentation, emphasizing the need for timely intervention. Key risk factors, including hypertension, diabetes, and chemotherapy, were identified as contributors to BRONJ development, underlining the importance of individualized treatment plans. Future research should refine diagnostic and preventive strategies, improve early intervention, and develop better treatment options, particularly for patients with complex comorbidities.

## Figures and Tables

**Table 1:** Distribution of patients based on secondary diagnosis.

Secondary Diagnosis		n	%
	Cancer	60	83.3
	Osteoporosis	11	15.3
	Cancer and osteoporosis	1	1.4
Detailed secondary diagnosis	Digestive cancer	1	1.4
	Genital cancer, thyroid and thymus cancer	1	1.4
	Multiple myeloma	4	5.6
	Prostate cancer	22	30.6
	Pulmonary cancer	6	8.3
	Renal cancer	5	6.9
	Breast cancer	21	29.2
	Osteoporosis	11	15.3
	Multiple myeloma and osteoporosis	1	1.4

**Table 2:** Distribution of risk factors.

Anemia	Absent	70	97.2
	Present	2	2.8
Chemotherapy	Absent	57	79.2
	Present	15	20.8
Diabetes	Absent	56	77.8
	Present	16	22.2
Arterial hypertension	Absent	44	61.1
	Present	28	38.9
Periodontal Disease	Absent	70	97.2
	Present	2	2.8

## Data Availability

The original contributions presented in this study are included in the article. Further inquiries can be directed to the corresponding authors.
